# Effects of indoor air movement and ambient temperature on mosquito (*Anopheles gambiae*) behaviour around bed nets: implications for malaria prevention initiatives

**DOI:** 10.1186/s12936-021-03957-y

**Published:** 2021-10-30

**Authors:** James F. Sutcliffe, Shaoman Yin

**Affiliations:** 1 Dept. Biology, Trent University, Peterborough, ON Canada; 2 Entomology Branch, US Centers for Disease Control and Prevention, Atlanta, GA USA; 3 CDC Foundation, Atlanta, GA USA

## Abstract

**Background:**

Until recently, relatively little research has been done on how mosquitoes behave around the occupied bed net in the indoor environment. This has been partly remedied in the last few years through laboratory and field studies, most of these using video methods and mosquito flight tracking. Despite these recent advances, understanding of the mosquito-bed net environment system, and the principles that underlie mosquito behaviour within it, is limited. This project aimed to further understand this system by studying the effects of gently moving air (such as might be introduced through room design to make the indoor environment more comfortable and conducive to ITN use) and warmer vs. cooler ambient conditions on mosquito activity around ITNs and other bed nets.

**Methods:**

The activity of colonized female *Anopheles gambiae* around an occupied untreated bed net set up in a mosquito-proof tent in a large laboratory space was recorded under different ambient conditions using a laser detection-video recording system. Conditions tested were ‘cool’ (23–25 °C) and ‘warm’ (27–30 °C) air temperatures and the presence or absence of a cross-flow produced by a small central processing unit (CPU) fan pointed at the side of the net so that it produced a ‘low-’ or ‘high-’ speed cross-draught (approx. 0.1 and 0.4 m/s, respectively). Near-net activity in recordings was measured using video image analysis.

**Results:**

In cool, still air conditions, more than 80% of near-net activity by *An. gambiae* occurred on the net roof. Introduction of the low-speed or high-speed cross-draught resulted in an almost total drop off in roof activity within 1 to 2 min and, in the case of the high-speed cross-draught, a complementary increase in activity on the net side. In warm, still conditions, near-net activity appeared to be lower overall than in cool, still air conditions and to be relatively less focussed on the roof. Introduction of the high-speed cross-draught in warm conditions resulted in a decrease in roof activity and increase in side activity though neither effect was statistically significant.

**Conclusions:**

Results are interpreted in terms of the flow of the stimulatory odour plume produced by the net occupant which, consistent with established principles of fluid dynamics, appears to rise quickly and remain more intact above the net occupant in cool, still air than in warm, still air. Cross-draught effects are ascribed to the changes they cause in the flow of the host odour plume as opposed to mosquito flight directly. The implications of these results for house designs that promote indoor air movement, on bed net design, and on other vector control measures are discussed. How mosquitoes approach a net is influenced both by indoor temperature and ventilation and their interaction. This system is in need of further study.

**Supplementary Information:**

The online version contains supplementary material available at 10.1186/s12936-021-03957-y.

## Background

Insecticide-treated bed nets (ITNs) are one of the mainstays in the campaign to reduce global malaria. According to the 2020 World Malaria Report [1], more than 2.2 billion ITNs have been distributed to households in virtually all parts of the malaria endemic world since 2000, with 86% of these in sub-Saharan Africa. ITNs, along with other vector control measures such as indoor residual spraying, are credited with preventing over 1.5 billion malaria cases and 76 million malaria deaths since 2000 [2]. Despite the success of ITNs and other approaches in reducing the malaria burden, 229 million malaria cases and 409,000 malaria deaths occurred in 2019. New methods of vector control and the refinement of established methods are needed to continue to reduce malaria [3].

Host-seeking in mosquitoes has been studied extensively in the field and in the laboratory. The interactions of mosquitoes with the various stimuli emitted by prospective hosts and with features of the environment have been reviewed many times [4–6] providing a general framework into which the mosquito’s attempt to find a human sleeping under a bed net can be fitted. In this framework, the occupied ITN is a baited mosquito trap that, like other traps, uses the mosquito’s host-seeking behaviours to be effective. Host receptivity in mosquitoes, like other blood feeding Diptera, is controlled by a range of biotic and abiotic factors (see [5] for a summary). The ITN strategy first relies on the propensity of certain *Anopheles* mosquitoes to enter human habitations to rest during the day or in the evening [7, 8]. Once receptive, many anopheline mosquitoes become activated into host-seeking by the elevated CO_2_ levels [6, 9] resulting from the presence of householders returning from daytime activities to retire for the night. Mosquitoes then orient to sleeper-originating stimuli such as breath and skin odours, heat and moisture [5, 6] but, in the process of following this trail, are blocked by the net barrier. Even if mosquitoes are not quickly incapacitated by the insecticide in the ITN, they are physically blocked from biting the occupant. Operational aspects of ITNs have been studied extensively in accordance with World Health Organization (WHO) guidelines [10] the aims of which are to provide clear definitions and standards for ITNs and a uniform set of procedures to measure various physical (e.g. burst strength, field durability), chemical (insecticide levels, wash resistance) and entomological (mosquito mortality, blood feeding inhibition) characteristics of candidate ITNs in lab studies and small and large-scale field trials. While WHO guidelines are important for establishing quality control, studies based on operational objectives provide little insight into the actual interactions of mosquitoes with the baited ITN ‘trap’ and the mechanisms that underlie them. Without such an understanding, it is difficult to fully explain or predict mosquito behaviour around bed nets within current models of blood feeder host-seeking.

Although insights are still limited, the past decade has seen significant progress in this area of study. In 2013, Lynd and McCall [11] reported using a sticky net, and in 2014 Sutcliffe and Yin [12] reported using sticky panels arrayed on a net, in laboratory studies to capture mosquitoes flying close to different parts of occupied bed nets. This provided the first experimental confirmation of anecdotal accounts of mosquitoes concentrating on the upper parts of nets [13]. Both studies found that up to 80% of captures of colonized *Anopheles gambiae* were on the bed net roof with a secondary focus on the lower part of the net sides and very few on the upper sides. Sutcliffe and Yin [12] further showed that colonized *Anopheles albimanus* is even more strongly roof-oriented (approx. 90% of captures) than *An. gambiae* and appear minimally on the net sides at any level, thus establishing that response patterns are not necessarily the same for all species. Subsequent studies have employed videos in different ways to investigate the mosquito-net interaction. Sutcliffe and Colborn [14] video recorded mosquitoes in simulated damaged bed nets to determine the mechanics and probabilities of net entry through holes of different sizes and orientations in the net sides or roof. Parker et al. [15, 16] and Angarita-Jaimes et al. [17] reported the development and use of an infrared tracking system to record flight tracks of mosquitoes in the space around occupied bed nets. Using this method, it has been possible to confirm and quantify the previously reported roof-bias and to resolve several characteristic flight behaviours around the net. Infrared flight tracking has also been used to compare colonized and wild mosquito behaviours around bed nets and to quantify the amount of treated net contact required for knockdown [16]. Another system, using lasers and video to detect and record near-net approaches by *An. gambiae* has been used to develop a tool to predict net entry risk based on location, size and shape of net holes [18]. This same system has also been used successfully in the field in Guatemala to compare near-net behaviours of wild-caught and colonized *An. albimanus* (Sutcliffe et al*.*, unpublished).

While progress in filling in the details of the behavioural framework of mosquitoes around bed nets has been made in the last few years, studies to date have been done under limited sets of conditions in the laboratory or field. Notably, these experiments have all been done in still air and, though they have been done at a variety of ambient temperatures and humidity levels, these were usually what the circumstances presented and have not been altered systematically to represent more fully the range of conditions under which ITNs are used. This study attempts to address some of these knowledge gaps by using the laser-detection video recording system previously described [18] to investigate the effects of air movements and different ambient temperatures (and their interactions) on near-net mosquito behaviour.

## Methods

### Experimental insects

Mosquitoes for this study were from a colony of *An. gambiae *sensu stricto (G3 strain) maintained by the Malaria Branch at the Centers for Disease Control and Prevention (CDC) in Atlanta, Georgia, USA. Colonized larvae, pupae and adults were reared at 28 °C on a 12 h:12 h light:dark cycle with a 30 min artificial sunrise and sunset. Colony adults emerged directly into 4 L cylindrical cardboard cages and were provided with 10% corn syrup in water ad libitum.

### Experimental setting and recording system

**Fig. 1:**
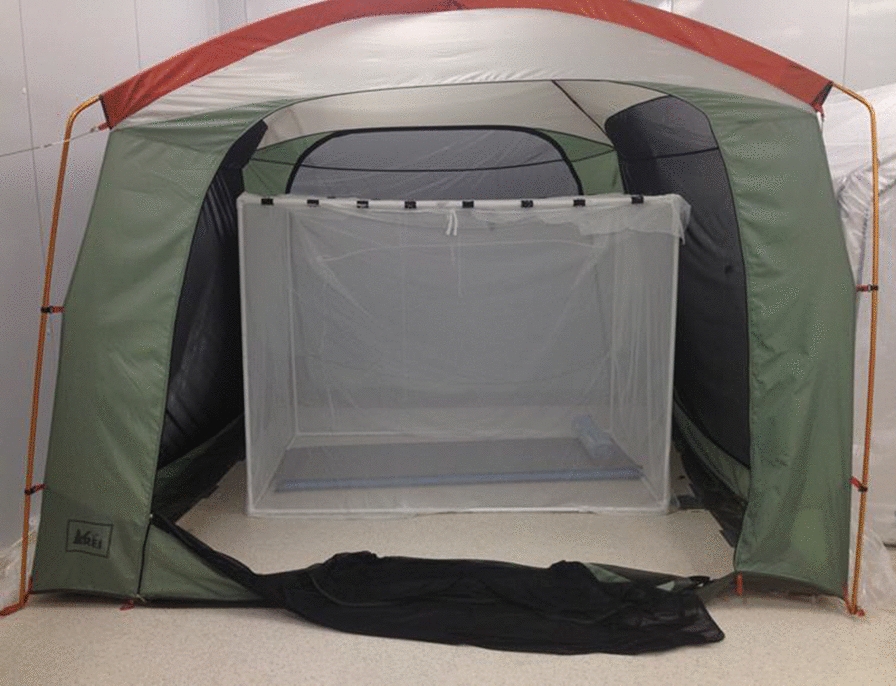
REI^®^ tent with untreated bed net fastened to a PVC frame with paper clamps. Cameras and CPU fan not present

All recording sessions were done in one of two large windowless experimental spaces (approx. 10 m × 5 m × 5 m high) in the insectary facilities of the Entomology Branch of the CDC in Atlanta. The sessions were conducted using an untreated polyester bed net erected on a 180 cm × 130 cm × 150 cm high PVC tube frame in a mosquito-proofed 3 m × 3 m × 2.1 m tall REI^®^ screen house (‘tent’) (REI catalog #794-289-0018, Fig. 1) in each large room.

**Fig. 2:**
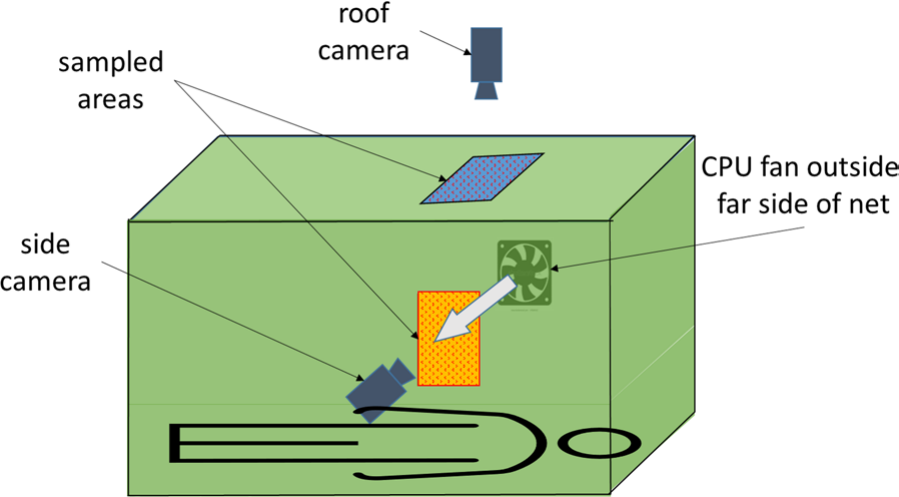
Diagrammatic depiction of occupied bed net illustrating cameras and sampled areas on the roof and side. Note that the CPU fan is outside the net and several centimetres from the far side mesh. Arrow represents cross-draught when fan is turned on. Tent, lasers and laser fields left out for simplicity. See Sutcliffe et al. [18] for details of laser placement

Recording sessions used Noldus Media Recorder^®^ software (version 2.1) and two Axis IP cameras (Axis Corporation, Sweden, Model M1144-L) operating at 15fps aimed at selected 30 cm × 60 cm sampling areas of the net (Fig. 2). Room lights were off during these sessions and near-net activity was detected by red line lasers (Apinex, 3 V, 5 mW, 650 nm, 90° fan angle) directed 1–1.5 cm above the sampling area on the roof of the net and across the sampling area on the net side. Each close approach to the net in a sampling area took a mosquito through a laser field creating a red flash that was recorded by the camera directed at that area (Additional file 1). See the method described by Sutcliffe, Ji and Yin [18] for additional details.

### Tent/net experimental conditions

Conditions inside the tent/net space during recording sessions were adjusted with respect to temperature and cross-draught speed so that mosquito responses around the occupied net to the presence and absence of a cross-draught could be observed under three different combinations of ambient temperature and air movement in the form of cross-draughts. These were: (A) cool temperature and low-speed cross-draught; (B) cool temperature and high-speed cross-draught and (C) warm temperature and high-speed cross-draught (see Table 1).

**Table 1:** Average activity measurements in each leg of each comparison for cool conditions (23–25 °C) with low- and high-speed cross-draught and warm conditions (27–30 °C) with high-speed cross-draught

	Recording session	Cross-draught	Average adj. appearances/min	
			Roof	Side
Cool conditions	1	Off	39.5	1.5
		On (low)	0.0	1.6
	2a	Off	179.2	1.9
		On (low)	6.2	5.9
	2b	Off	59.2	0.5
		On (low)	9.5	31.6
	3a	Off	276.3	0.1
		On (low)	37.4	19.8
	3b	Off	293.3	0.1
		On (low)	9.6	3.0
	4a	Off	292.8	0.3
		On (high)	2.9	125.8
	4b	Off	105.2	7.9
		On (high)	17.6	155.2
	5a	Off	121.8	0.2
		On (high)	8.4	220.9
	5b	off	229.4	16.7
		On (high)	13.8	163.1
Warm conditions	6a	Off	7.8	16.7
		On (high)	0.8	27.4
	6b	Off	10.8	19.8
		On (high)	4.4	7.0
	7a	Off	24.2	3.9
		On (high)	0.3	1.4
	7b	Off	7.2	9.7
		On (high)	1.0	2.4
	8a	Off	16.4	2.4
		On (high)	0.8	40.5
	8b	Off	38.3	20.9
		On (high)	2.1	49.3
	9a	Off	40.7	7.6
		On (high)	0.7	31.7
	9b	Off	55.8	33.1
		On (high)	6.7	99.6
	10	Off	8.1	4.0
		On (high)	0.3	83.0
	11a	Off	22.4	12.1
		On (high)	3.3	284.0
	11b	Off	195.0	20.0
		On (high)	59.4	21.8

#### Temperature and humidity

In preparation for each recording session, heating was used (if necessary) to adjust ambient temperature in the experimental room to achieve nominally ‘cool’ (23–25 °C) or ‘warm’ conditions (27–30 °C) such as might be experienced in houses overnight in the tropics. The cool range was the normal ambient range of the experimental space while the warm range was chosen as one that could readily be sustained by the chamber heating system and that corresponds to overnight indoor temperatures in many malaria endemic areas [19, 20]. Additional moisture was added with humidifiers in warm conditions to help prevent mosquito desiccation, but humidity was not an experimental treatment.

#### Cross-draughts

Cross-draughts through the net were produced with a tripod-mounted 120 mm diameter central processing unit (CPU) cooling fan positioned approximately 70 cm above the floor of the tent and 30–50 cm from the side of the net. The fan, which could be turned on and off from inside the net, was directed at right angles to the net side at roughly its mid-level above the net occupant’s torso and directly across from the sampling area on the other side of the net (Fig. 2). The cross-draught speed was regulated by the number of 50 cm × 50 cm mesh pieces that were placed across the CPU fan outlet. The resulting in-net cross-draught speeds were measured and calibrated using video to determine the cross-net transit time of repeated puffs of fine powder introduced at the upwind side of a bed net erected inside the REI tent. A speed of approximately 0.1–0.2 m/s was chosen for the low-speed cross-draught (LSCD) because it resulted in positive but barely perceptible air movement on the outside of the downwind side of the net (after passing through all intervening mesh layers). A speed of approx. 0.3–0.4 m/s was chosen for the high-speed cross-draught (HSCD) because it was clearly perceptible outside the downwind side of the net but did not interfere with mosquito flight. The fan-off condition is reported herein as ‘still air’ (i.e. cross-draught speed = 0 m/s) since air movement in the rooms overall was slight and did not have a measurable effect inside the tent.

### Recording session procedures

In the morning before the day’s recording session a cage of mosquitoes (4 to 8 days old, not previously blood fed) was taken from the general colony and held without access to sugar water until approximately 30 min before the late afternoon start of the session. Between 100 and 150 female mosquitoes were then removed with a HEPA-filtered aspirator and put into screw-top polystyrene vials (approximately 3 cm wide × 8 cm tall, up to 50 mosquitoes per vial). To help ensure the mosquitoes would seek a blood meal, females were drawn from those that landed on, and tried to probe through, the colony cage’s mesh sleeve when it was draped across a bowl of warm water. In preparation for release, vials, with lids loosened, were placed on the floor of the tent housing the bed net on the side away from the tent entrance.

After the line lasers had been turned on and their alignments checked, the recording system was started, the room lights were turned off and the experimenter, wearing shorts, a short-sleeved shirt and socks, but no shoes, entered the tent, closed it and entered the net. The experimenter then released the mosquitoes by reaching from inside the net to knock the loosely capped vials over. After securing the net, the experimenter laid on an air mattress facing upward and remained as still as reasonably possible throughout the session.

Each recording session began with a 5 to 10-min ‘fan off’ period to allow mosquitoes to acclimate post-release and for any minor air disturbance created by the experimenter’s movements to dissipate. This was followed by a timed still air period in which the fan was off, followed by a timed cross-draught period in which the fan was on. These two periods formed the two approximately 12–15 min ‘legs’ of a comparison contrasting mosquito behaviour with and without a cross-draught. In most sessions this sequence was performed twice. At the end of the session, the experimenter exited the net, shut down the lasers, exited the tent and shut off the recording system. Any surviving mosquitoes were cleared from the tent the next morning using a HEPA-filtered mouth aspirator. Mosquitoes were not re-used.

In addition to sessions with an occupied bed net, two control sessions (bed net unoccupied) were run, one with a still air-LSCD comparison consisting of 20-min legs of each condition and the other with a still air-HSCD comparison consisting of 5-min legs of each condition.

### Analysis of videos and statistical procedures

Videos were analysed using Noldus Ethovision^®^ (ver. 10.1) motion tracking software. The approximately 60 cm × 30 cm sampling ‘arena’ on the net roof had its shorter ‘Y’-axis parallel to the net’s length while the arena on the side had its shorter Y-axis oriented horizontally. Near-net approaches in the arenas were detected by Ethovision as red flashes when mosquitoes flew through the laser fields (Additional file 2). All analyses were done in the ‘differencing’ mode of Ethovision with the subject set as ‘brighter’ than the background, contrast sensitivity was 30 (arrived at through previous trial and error) and the minimum subject size was 30 pixels. No maximum subject size was set but large outlier detections in each recording (resulting mostly from light interference) were manually removed before further analysis. Each red flash signifying near-net activity was designated an ‘appearance’.

To arrive at equivalent near-net activity values across all sessions, raw appearance numbers were adjusted to account for leg duration, Ethovision efficiency (which varied with video quality and CPU efficiency of the computer used for the Ethovision analysis) and number of mosquitoes released (which varied due to fluctuations in the colony) to yield ‘adjusted appearances per minute’ (adj. apps/min) in the arena for each leg of each comparison (Table 1).

Each leg of each comparison in the three ambient temperature—cross-draught speed combinations was assigned to one of four conditions: (1) fan off, roof location, (2) fan off, side location, (3) fan on, roof location and (4) fan on, side location. The generalized linear model (GLM) procedure was used to regress the adj. apps/min within each of the ambient temperature—cross-draught speed combinations with the quasi-likelihood estimation considering the skewed distribution of the measurements. This resulted in six pairwise comparisons in each of the three combinations. Statistical differences of comparisons of adj. apps/min of pairs of conditions were assessed with the Bonferroni–Holm adjustment for multiple post-hoc comparisons [21]. R statistical software and an R package “mulcomp” were used in the analyses [22]. The GLM procedure was also used to compare adj. apps/min between combined cool and warm legs at the roof location and, separately, at the side location.

## Results

Control sessions, in which the bed net was unoccupied, revealed virtually no activity at either roof or side sampling sites in still air or with either low-speed or high-speed cross-draught.

Overall mosquito activity levels when the bed net was occupied varied considerably between legs done under the same conditions and between cool and warm conditions (Table 1). For example, activity in still air, the ‘default’ state, under cool conditions ranged from 39.5 to 293.3 adj. apps/min on the roof and 0.1 to 16.7 adj. apps/min on the side. Under warm conditions, the corresponding ranges were 7.2 to 195.0 adj. apps/min on the roof and 2.4 to 33.1 adj. apps/min on the side.

### Cool conditions

**Fig. 3:**
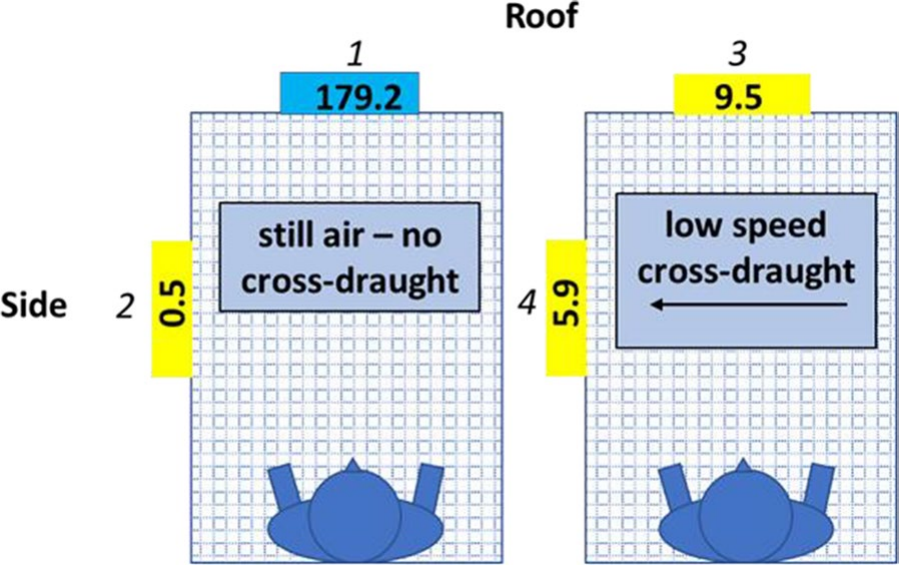
Near-net activity median values for comparisons done in cool conditions with and without a low-speed cross-draught. Cross-hatched rectangles represent end-on views of the occupied bed net. The supine occupant is depicted as if seen from the top of the head. Median roof and side near-net activity is displayed in coloured boxes for still air (left rectangle) and for the presence of a cross-draught (right rectangle). Boxes for locations at which activity levels do not differ statistically share the same colour. Numbers in italics above or beside the median values correspond to comparisons detailed in Table 2

**Fig. 4:**
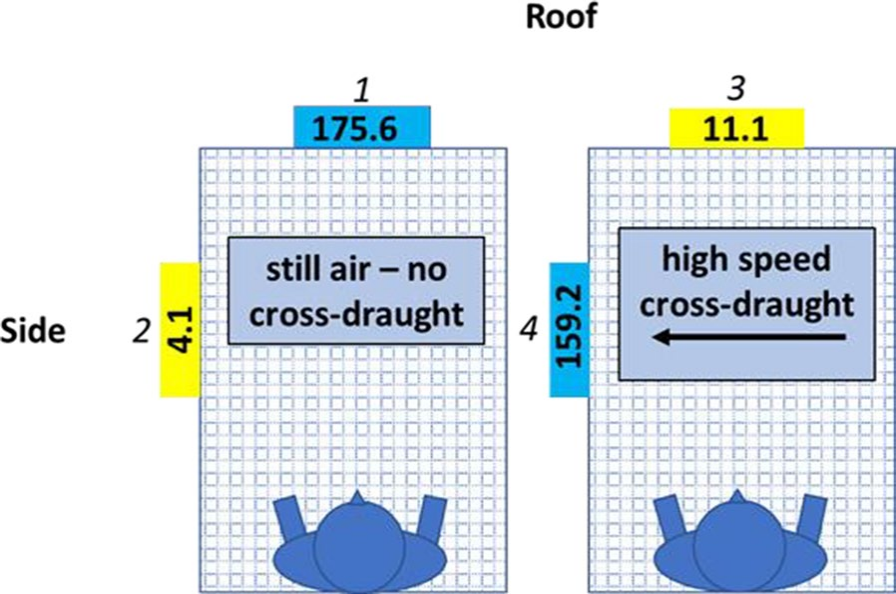
Near-net activity median values for comparisons done in cool conditions with and without a high-speed cross-draught. See Fig. 3 for an explanation of figure features. Numbers in italics above or beside the median values correspond to comparisons detailed in Table 3

Average roof activity in the nine still air legs in cool conditions was more than 50 times greater than side activity under the same conditions (Table 1). In the five cool comparisons involving LSCDs, still air median activity on the roof was 179.2 adj. apps/min compared to 0.5 adj. apps/min on the side (p < 0.001, Fig. 3, Table 2) and in the four cool comparisons involving the HSCDs, still air median roof activity was 175.6 adj. apps/min and median side activity was 4.1 adj. apps/min (p < 0.001, Fig. 4, Table 3).

**Table 2:** Results for cool, low-speed cross-draught comparisons

Comparison	Estimate (adj. apps/min)	95% CI of estimate		z value	p value
		Lower	Upper		
1–2	168.7	74.6	262.8	5.33	< .000002
1–3	156.9	62.9	251.1	4.959	< .00002
1–4	157.1	63.1	251.2	4.964	< .00002
2–3	− 11.7	− 11.7	− 105.8	− 0.37	ns
2–4	− 11.6	− 105.7	82.6	− 0.365	ns
3–4	0.16	− 93.9	94.3	0.005	ns

**Table 3:** Results for cool, high-speed cross-draught comparisons

Comparison	Estimate (adj. apps/min)	95% CI of estimate		z value	p value
		Lower	Upper		
1–2	181	75.8	286.4	5.116	< .00006
1–3	176.6	71.4	281.8	4.992	< .00002
1–4	21.1	− 84.2	126.3	0.595	ns
2–3	− 4.4	− 109.6	100.8	− 0.124	ns
2–4	− 159.9	− 260.8	− 50.4	− 4.521	< .00009
3–4	− 155.6	− 260.8	− 50.4	− 4.397	< .00015

Initiation of cross-draughts at either speed in cool comparisons resulted in rapid virtual cessation of roof activity. Compared to in still air, median roof activity in the LSCD legs dropped by almost 95% to 9.5 adj. apps/min (p < 0.001, Fig. 3, Table 2) and by over 90% to 11.1 adj. apps/min (p < 0.001, Fig. 4, Table 3) in HSCD legs.

While cross-draughts at both speeds reduced roof activity to very low levels, cross-draught speed had different effects on side activity. Median activity in the presence of the LSCD increased by more than a factor of 10 to 5.9 adj. apps/min on the net side but this difference was not statistically significant nor was the greater side activity in the presence of the LSCD significantly different from roof activity without the LSCD (Fig. 3, Table 2). In contrast, side activity in the presence of the HSCD increased almost 40-fold to 159.2 adj. apps/min which was significantly greater than side activity in still air (4.1 adj. apps/min) (p < 0.0009, Fig. 4, Table 3) and roof activity in the presence of the HSCD (11.1 adj. apps/min) (p < 0.00015, Fig. 4, Table 3).

**Fig. 5:**
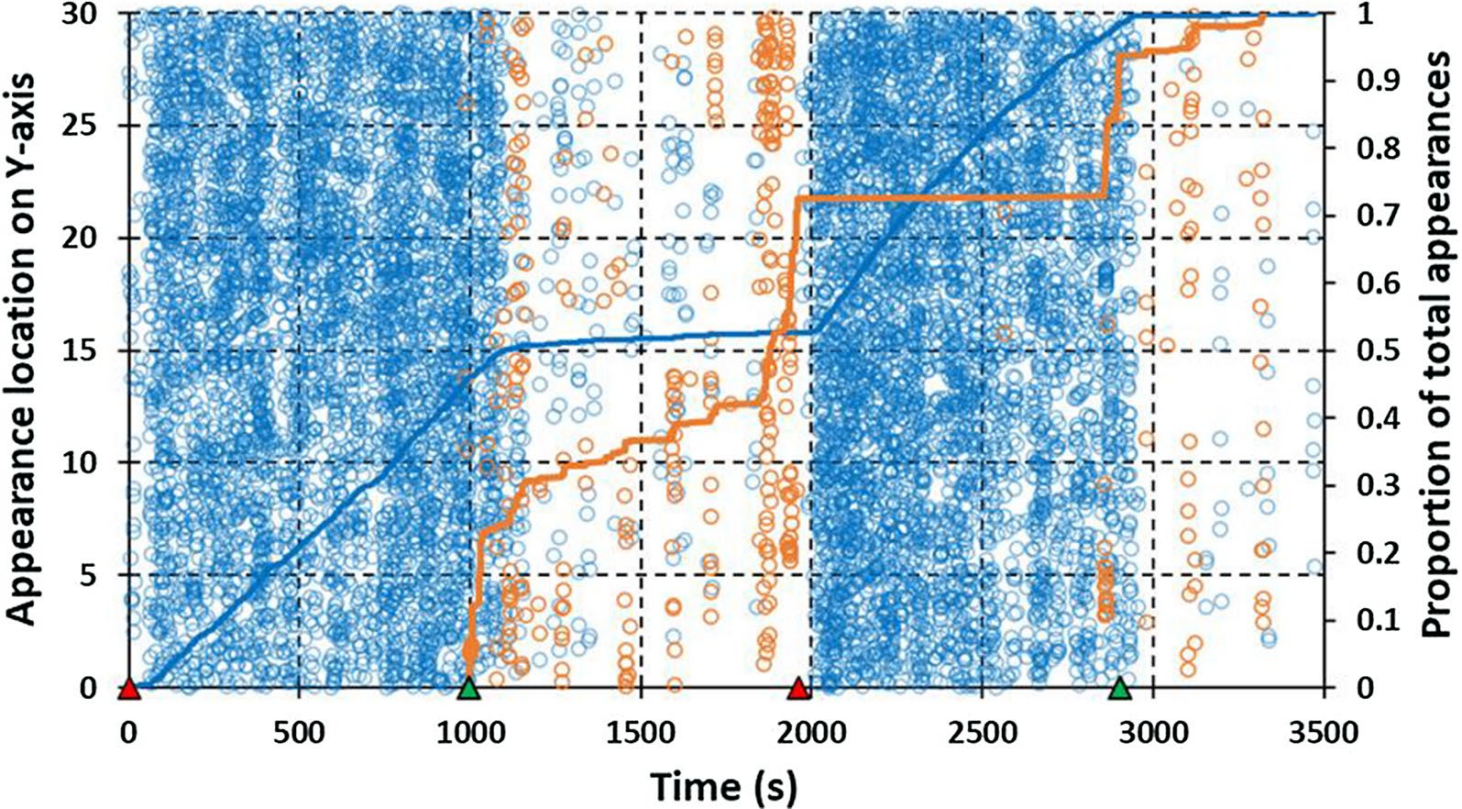
Plots of near-net activity in cool conditions in low-speed cross-draught session 3 (Table 1). Corresponding total adjusted appearances on the roof = 9720 and on the side = 361. Circular markers represent the Y-axis position of near-net mosquito appearances on the roof sampling area (blue) and the side sampling area (brown) plotted over the time course of the recording session. Density and patterning of markers provides a heat map impression of activity intensity and appearance ebbs and flows over time. Superimposed lines represent the cumulative proportion of appearances on the roof (blue) and the side (brown) over the time course of the recording session. Red triangle markers on the horizontal axis indicate the start of still air legs (fan off) and green markers indicate the start of cross-draught legs (fan on)

Mosquito appearances near the net plotted over time for the roof and side in session 3 (Fig. 5) illustrate typical patterns for still air-LSCD comparisons. Intense roof activity, normally seen in still air, reduced to almost zero within 1–2 min of the two initiations of the LSCD at 998 and 2903 s and remained very low while the fan remained on. Stoppage of the LSCD at 1961s, re-establishing still air conditions, was followed by restoration of roof activity within 1–2 min to near previous levels. Side activity in still air was very low and increased within 1–2 min of initiation of the LSCD, but to much lower levels than previously seen on the roof and decreased again within 1–2 min of fan shut-off.

**Fig. 6:**
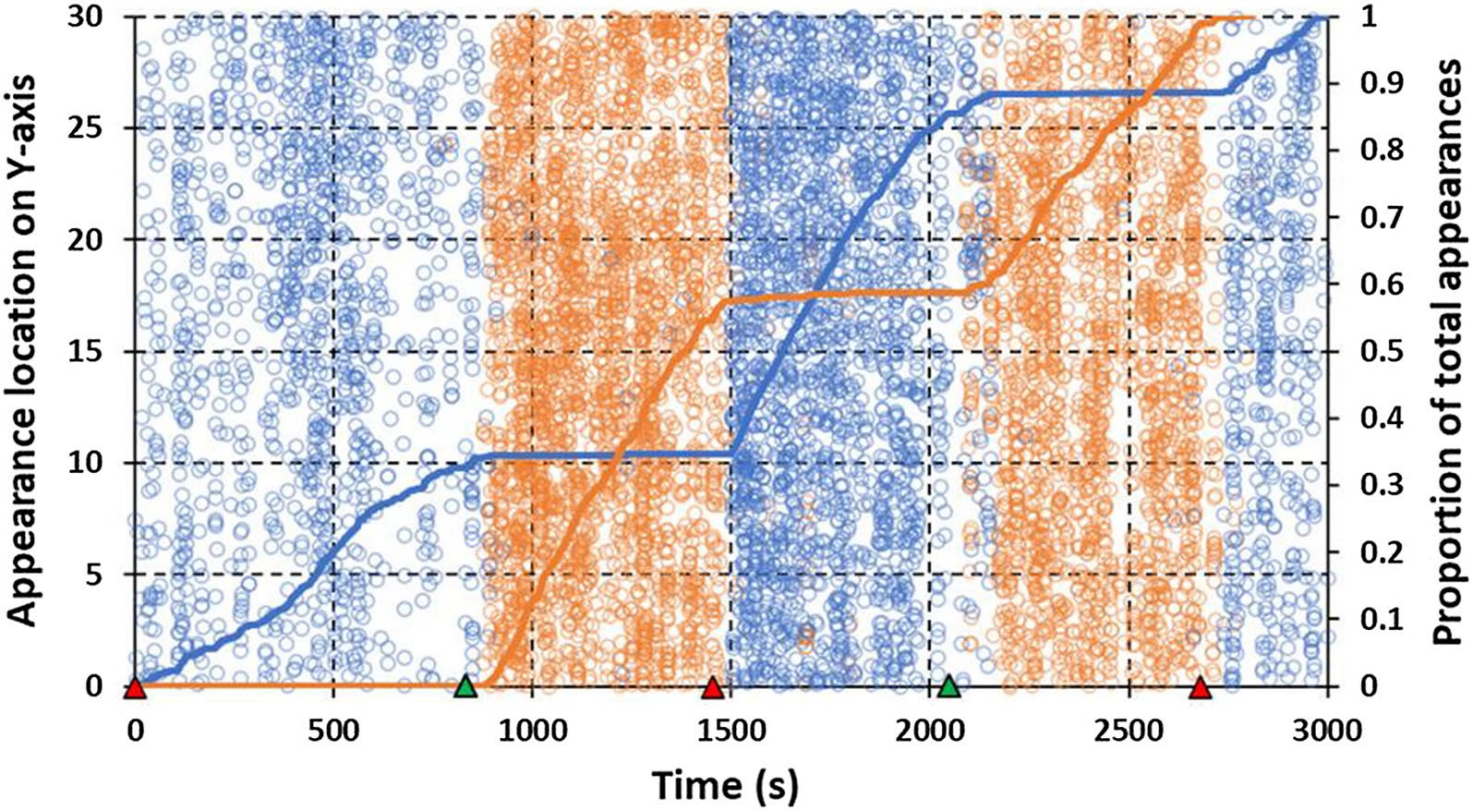
Plots of near-net activity in cool conditions in high-speed cross-draught session 5 (Table 1). Corresponding total adjusted appearances on the roof = 3717 and on the side = 3996. See Fig. 5 for an explanation of figure features

Appearances plotted over time for a typical still air-HSCD series are illustrated by session 5 (Fig. 6). Patterns on the roof were similar to still air-LSCD with clear drop-offs in activity in response to HSCD initiation at 832 s and 2080 s. On the other hand, side activity, which was again very low in the absence of the cross-draught, increased 10- to over 1000-fold in the HSCD. Roof and side activity in these comparisons closely complement each other in timing and are of similar intensity. As in the LSCD sessions, transitions from low to high (or vice versa) levels of activity when the HSCD was initiated (or ended) occurred within 1–2 min.

### Warm conditions

In still air there was significantly less activity in warm conditions on the roof than in cool conditions (estimate: 318.0, 95% confidence interval 208.6–427.4, z = 8.641, p << 0.001). No statistically significant difference in still air activity was detected on the side between cool and warm conditions. This is reflected by the fact that roof activity in still air warm conditions was greater than side activity in 8 of 11 comparisons but only by an overall factor of approximately threefold, compared to more than 50-fold in cool conditions (Table 1). This indicates that mosquito activity overall in warm conditions was more dispersed than in cool conditions.

**Fig. 7:**
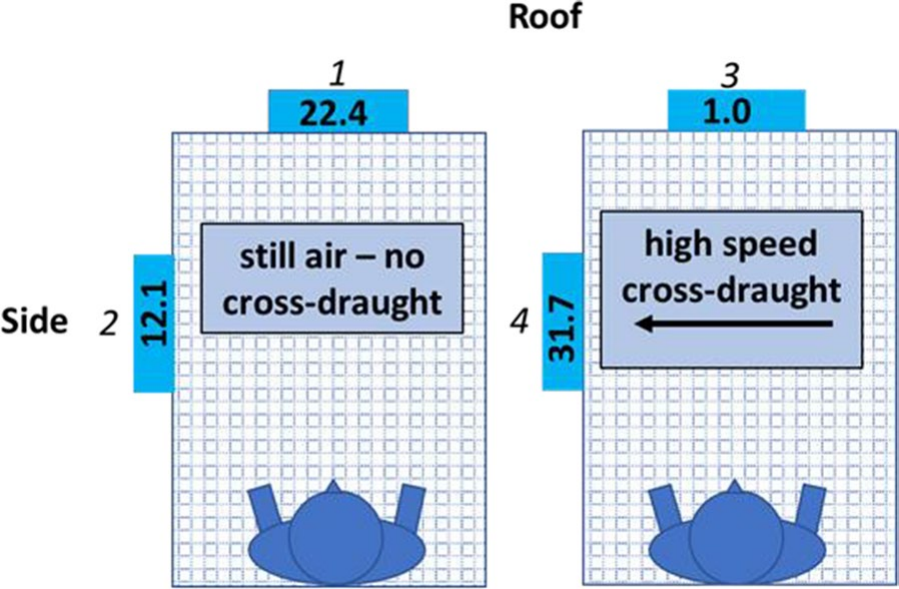
Near-net activity median values for comparisons done in warm conditions with and without a high-speed cross-draught. See Fig. 3 for an explanation of figure features. Numbers in italics above or beside the median values correspond to comparisons detailed in Table 4

Changes in activity between still air and cross-draught legs in warm conditions were less distinct in these sessions (Fig. 7) than in cool comparisons. Median activity at the roof sampling site in warm conditions decreased from 22.4 adj. apps/min in still air to 1.0 adj. apps/min when the HSCD was present and median side activity increased from 12.1 to 33.1 adj. apps/min in the presence of the HSCD; however, none of these effects was statistically significant (Table 4).

**Table 4:** Results for warm, high-speed cross-draught comparisons

Comparison	Estimate (adj. apps/min)	95% CI of estimate		z value	p value
		Lower	Upper		
1–2	25.1	− 38.3	88.6	1.178	ns
1–3	31.5	− 31.9	95	1.478	ns
1–4	− 16.9	− 80.3	46.6	0.79	ns
2–3	6.4	− 57	69.9	0.3	ns
2–3	− 42	− 105.4	21.5	1.968	ns
3–4	− 48.4	− 111.8	15.1	− 2.268	ns

**Fig. 8:**
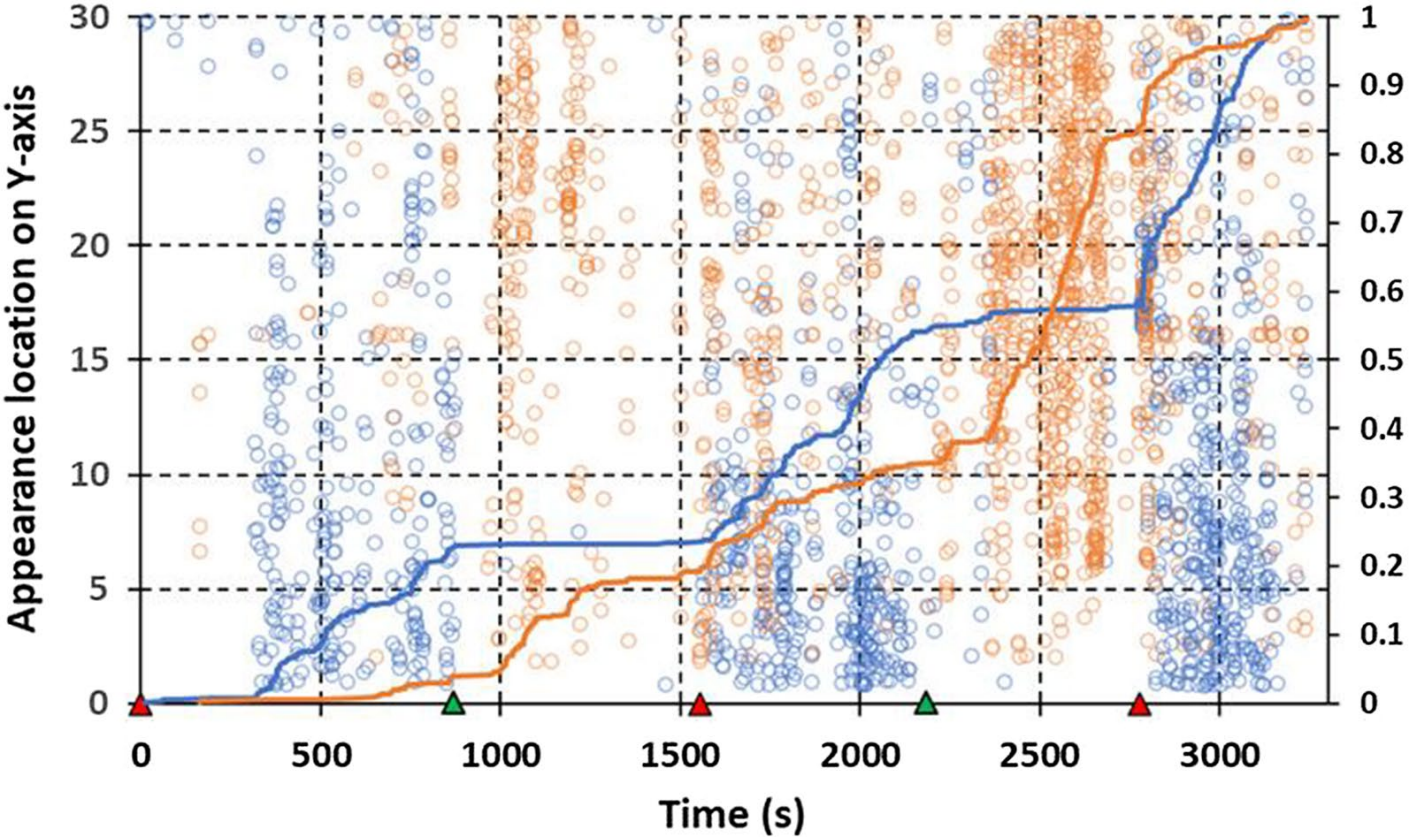
Plots of near-net activity in warm conditions in high-speed cross-draught session 9 (Table 1). Corresponding total adjusted appearances on the roof = 1029 and on the side = 1701. See Fig. 5 for an explanation of figure features

Several features that characterize warm sessions are illustrated in appearance vs. time plots of session 9 (Fig. 8). As in almost all cool sessions, initiation of the HSCD in warm conditions was followed by a large apparent drop in roof activity but the HSCD had relatively less effect on side activity in warm conditions. Indeed, though more numerous in the presence of the HSCD, appearances on the side in warm conditions tended to occur throughout the entire session and changes in rate of side appearances were less tied to transitions from still air to the HSCD, and vice versa.

## Discussion

### General

The absence of near-net activity in control sessions with no one in the bed net is consistent with similar work [11, 12, 15–18] and confirms that bed net-oriented behaviour seen in this study was due to stimuli emanating from the net occupant. While the methods used in this study cannot determine how much activity occurred beyond the observed areas of the unoccupied control nets, or in the tent enclosure as a whole, flight tracking studies of free flying mosquitoes [16] in a similar setting suggest that, though many mosquitoes were likely actively flying in the tent space, they made little or no sustained close contact with any part of the net and were not orienting toward it.

The presence of a net occupant was necessary for a significant amount of near-net activity irrespective of temperature conditions and presence or absence of a cross-draught. In still air legs, an average of approximately 97% of all cool session appearances occurred on the net roof. This compares favourably with several similar studies also done in still air [11, 14–18]. Still air appearances in warm air sessions were also predominantly on the roof though only by an average of approximately 66% (see below for further discussion).

Introduction of the cross-draught at either speed and in both cool and warm sessions resulted in marked reductions, in many cases virtually to zero, of near-net activity at the roof sampling area. Several points support that this was the result of changes in the mosquitoes’ behavioural responses as patterns of host-related cues shifted due to the cross-draughts, and not of direct physical interference of the cross-draughts with mosquito flight: (1) airflows at the speeds used (0.1 to 0.4 m/s) are not strong enough to perturb flight greatly since mosquitoes orient in flight at wind speeds in excess of 1 m/s [23–25]; (2) activity changes on the roof (and sides) following cross-draught start-up (and cessation) occurred gradually over 1 to 2 min rather than abruptly as would have been the case had the mosquitoes simply been blown away; and (3) fan placement part way down the net side ensured that cross-draughts were at the mid-level of the net and did not directly contact mosquitoes on the roof or above it.

### Cool conditions

**Fig. 9:**
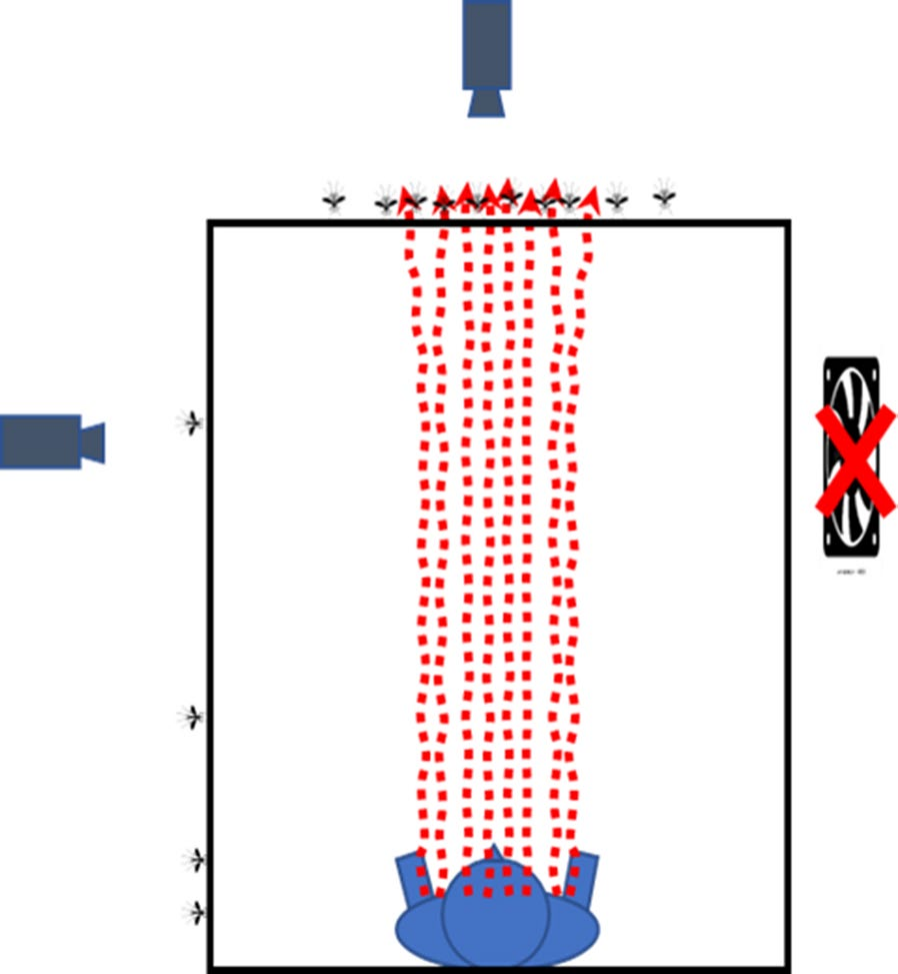
Diagrammatic depiction of the possible effects on mosquito near-net behaviour seen from the head end of the occupied bed net of still air in cool conditions. Mosquitoes’ near-net activity occurs largely on the net roof under the unperturbed influence of the rising odour plume. CPU fan is off. Cameras are above and to the left of the net, CPU fan is to the right of the net. Note that this diagram is meant to illustrate possible influences on mosquitoes and is not meant to capture the complexities of shape and internal dynamics of actual plumes

Massing of mosquito activity on the net roof indicates that stimuli from the net occupant (likely body and breath odours and moisture) rose upward as a warm convective plume through the roof under cool, still air conditions, but did not extend outward much, if at all, since there was very little still air activity on the sides. This is consistent with a chimney effect produced by the net sides and with modelling which shows that the heat-driven plume from a recumbent person at an ambient temperature of 24 °C, rises most strongly above the torso area reaching a maximum speed of approximately 0.23 m/s at a height of more than 1.8 m [26]. This means the plume would still have been moving upward at maximum speed when it hit the underside of the net roof approximately 1.5 m above the net occupant. Given the fact that the net mesh represents a partial barrier to air flow, the plume would likely have hit the underside of the roof and slowed down by about 50% as it passed through [19] but it would still have been moving upward at about 0.12 m/s above the net thus providing host-orienting cues to mosquitoes in the vicinity (see Fig. 9 for a diagrammatic depiction).

In the experimental tents after mosquitoes were released, elevated CO_2_ levels from the net occupant would likely have activated them into a host-seeking mode of behaviour [6, 9] resulting in unoriented, searching flight and eventual contact (for at least some of them) with the rising odour plume from the sleeper. Once in the plume, there are several mechanisms that could have provided information to guide mosquitoes toward its source. Temperature gradients or odour concentration gradients are both considered possibilities for host orientation at short range [27], although this may not apply in the current setting since turbulence introduced by the plume’s passage through the net might disrupt clear gradients. Positive anemotaxis using the optomotor response [28] is also a possibility since, even though recording sessions were done in darkness, stray light from computer equipment in the room and light leaking around the door may have provided enough illumination to allow this. This is especially plausible in the case of *An. gambiae* which can detect objects in very low light intensities (e.g. levels equivalent to 10 times starlight or less) [29]. Mosquitoes flying above the net may also have been able to use gravity to guide them down the plume although this mechanism cannot account for mosquitoes appearing at the side of the net in the presence of the cross-draught.

In normal circumstances, host cues would guide the mosquito to the sleeper but, in this case, progress is blocked by the bed net roof. Mosquitoes blocked in this way appeared to fly brief low meandering paths along the net roof surface in a pattern that is consistent with a behaviour called ‘bouncing’ by Parker et al. [16] in which host-seeking mosquitoes fly close to the net roof repeatedly coming into contact with it. Bouncing may come about as a result of the host-seeking mosquito repeatedly turning up-plume (upwind) only to be stopped by the mesh each time. Given this, the accumulation of large numbers of mosquitoes on the net roof in the host odour field such as seen here is presumably the result of behavioural responses evolved to help them maintain contact with favourable conditions (in this case the moist host plume). This appears to be what happened in studies by Spitzen et al. in which *An. gambiae* in a wind tunnel [30] and approaching an experimental hut eaves [31] reduced their flight speed and increased their turning rate, in what the latter calls an ‘exploration strategy’, when encountering host-associated odour.

The pattern of near-net mosquito activity changes observed on the roof when the LSCD or HSCD was present is consistent with cross-draught-caused perturbations to the cohesiveness and flow of the host odour plume. Even the gentle air flow of the LSCD appears to have been enough to disrupt and re-direct the rising plume so that little or none of it reached the observed area of the roof (Fig. 8). The sudden loss of the odour plume presumably results in decreased station-holding behaviours and bouncing and an increase in flight patterns functionally analogous to ‘casting’ in some moths [32] or downwind flight in tsetse [33] and some parasitoid wasps [34] all of which occur after losing contact with the orienting odour plume. These behaviours are thought to aid the insect regain plume contact (see Cardé and Gibson [27] for a summary of these behaviours). As wider searching patterns become more dominant in recording sessions due to loss of the plume, mosquitoes would have ranged further and further and eventually stopped appearing in observation area.

**Fig. 10:**
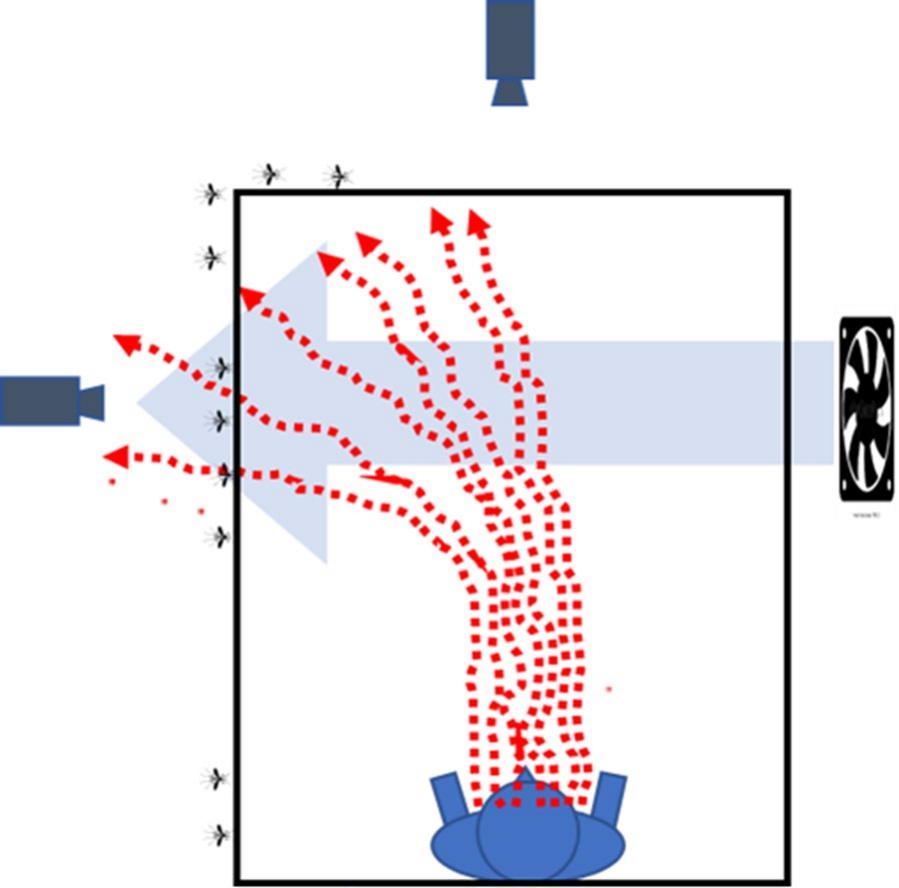
Diagrammatic depiction of the possible effects on mosquito near-net behaviour seen from the head end of the occupied bed net in cool conditions in the presence of the low-speed cross-draught. The gentle cross-draught disrupts plume shifting much of its influence away from the roof with the result that some near-net activity is displaced to the net side. See Fig. 9 for an explanation of figure features and limitations

The loss of mosquito activity on the roof when the cross-draughts were initiated was, to a greater or lesser extent depending on cross-draught speed, complemented by increased activity on the side. This is consistent with a partial re-direction of the plume by the cross-draughts. Allowing for a 0.23 m/s updraft from the net occupant in the sessions done in cool conditions [26], the plume could have been diverted by, on average, allowing for turbulence, approximately 20° from the vertical by the LSCD resulting in it being displaced roughly 30 cm downwind by the time it arrived at the roof. This is enough for the plume to largely miss the observed area of the roof though not enough to divert all but a small amount of it to the area the side where the camera was aimed. This may account for the slight increase in side activity in the LSCD (see Fig. 10 for a diagrammatic depiction). The remaining activity may have occurred in parts of the net that the cameras did not record.

**Fig. 11:**
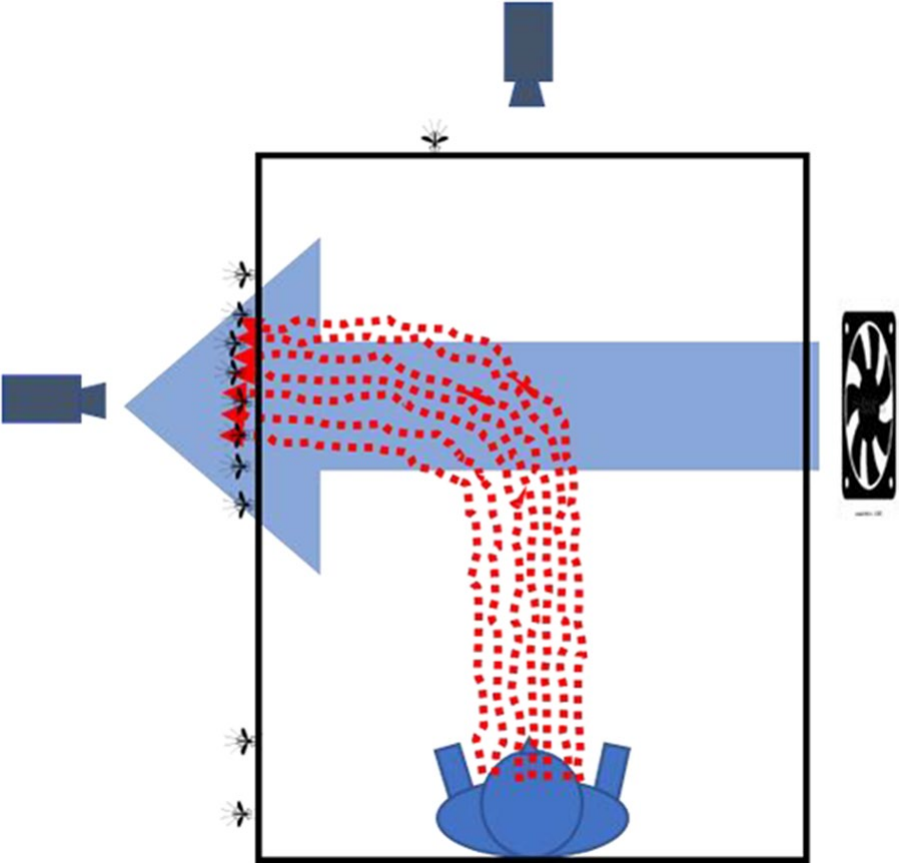
Diagrammatic depiction of the possible effects on mosquito near-net behaviour seen from the head end of the occupied bed net in cool conditions in the presence of the high-speed cross-draught. The strong cross-draught shifts the plume’s influence largely to the net side. See Fig. 9 for an explanation of figure features and limitations

For the HSCD legs, side activity was comparable to, and not significantly different from, roof activity during still air legs. This is consistent with the plume having been more completely diverted to the side by the faster moving cross-draught. The HSCD, at 0.4 m/s, would have diverted the plume by approximately 60°. At this angle, the plume would entirely miss the roof and a great deal of it would exit through the net side close to the area observed by the side camera (see Fig. 11 for a diagrammatic depiction).

In both LSCD and HSCD situations, activated mosquitoes, though not necessarily the same ones that appeared on the roof in still air legs, would presumably have responded to the plume exiting the side through the same plume-following mechanisms already suggested for those above the roof although gravity-directed flight would not apply.

### Warm conditions

Warm air is less dense and, therefore, supplies less buoyancy than cool air which means that the convective human odour plume should rise more and more slowly with increasing ambient temperature. Modelling the odour plume of a recumbent human at 30 °C shows the maximum upward speed to be approximately 0.14 m/s (compared to 0.23 m/s at 24 °C) and the plume height reduced to about 1.2 m before becoming attenuated and spreading out [26]. This is well below the roof of the bed net used in this study. This could explain the decreased roof activity in warm conditions since the plume, having run out of upward momentum but still needing to disperse, may have spilled out through the sides to a greater extent than in cool conditions. This is also consistent with the fact the greatest activity in warm sessions was at the side in the presence of the HSCD (Fig. 3) though this result was not statistically significant.

Other studies done at warmer temperatures (e.g. [15, 17]) do not show reduced roof activity to the extent this study does. Using the mid-range temperature from these studies (27 °C), the upward plume speed would have been, on average, 0.20 m/s and reached approximately 1.7 m before becoming attenuated [26]. This, combined with the fact that in these studies the net roof was much closer to the subject (due to a combination of net geometry needed for flight tracking and a mattress that elevated the net occupant approximately 18 cm above the floor), means that subject-to-net roof distance at the mid-point of the roof was approximately 60 cm—less than half the distance than in the present study. This means that mosquitoes on the roof were much closer to the occupant and the occupant’s plume which, even though less energetic than at 20 °C, would not have attenuated greatly by the time it got to the roof.

### Implications for tropical houses

Given the ability of a gentle 0.1 m/s LSCD to disrupt and re-direct the net occupant’s odour plume, it might be expected that a true upward plume flow reaching the net roof in real life bed net use situations would be rare. In this regard, it is noteworthy that in a study of households in Thailand, The Philippines, Tanzania and The Gambia, natural cross-draughts in bed nets were measured at well below 0.1 m/s in all but one case and, in many cases, below 0.03 m/s [19]. These would not be strong enough to deflect the plume from vertical significantly. If this is the case, most of the activity around real-life nets should, as in this study, also be on the roof in cooler nighttime conditions. However, based on these results, in warmer conditions, the strength and vertical reach of the plume should be reduced resulting (depending on the net height) in a less net roof-focussed pattern of activity. A number of studies in rural tropical homes show that overnight temperatures may vary greatly depending on location and time of year. Annual mean indoor temperatures at 2100 h were 33.1 °C, 29.4 °C, 27.5 °C and 26.4 °C in The Gambia, Tanzania, the Philippines and Thailand sites, respectively [19]. Nightly temperatures at these locations no doubt also varied through the year, as was found in Ouagadougou, Burkina Faso where in-house nighttime temperatures ranged from a maximum of 30.7 °C in May and April to 21.0 °C in December and January [20]. In both examples, temperatures span the range tested in this study suggesting that no single pattern of near-net mosquito behaviour is likely to apply everywhere or even in any given location the year round.

### Practical implications

These results have implications for several current vector control practices and products; in particular, for ITN design, bed net use practices and house design. Many ITN designs are currently reflected by the products on the WHO prequalified vector control products list [35]. These come in different materials, dimensions, and mesh sizes. Most incorporate a single insecticidal component in all panels while a few have more insecticide (with or without a synergist) in the roof panel and lesser amounts (with or without a synergist) in the side panels. Those with higher concentrations of insecticide plus a synergist in the roof panel, appear well-positioned to take advantage of the accepted notion of a strong roof orientation of malaria mosquitoes. However, such nets may not work as designed in warmer conditions where mosquito activity may not be focussed on the roof. The corollary to this is that the less vigorous plumes created by children and smaller adults might also result in less roof-oriented behaviour in host-seeking mosquitoes. This is partly confirmed by laboratory experiments in which catches of *An. gambiae* on sticky panels arrayed on the sides and roof of an untreated bed net were significantly less roof-oriented for the smallest subject (56 kg) than for either the other two larger subjects (75 kg and 84 kg) [12].

Of the many factors affecting bed net use in malaria-endemic areas, one of the most important negative influences is that they make users feel hot and uncomfortable [36, 37]. Through measurements taken outside and inside occupied nets, von Seidlein et al*.* [19] showed that this effect is not due to higher temperatures per se in nets but, rather, to attenuated airflows caused by the bed net that reduce evaporative cooling. To alleviate this and encourage greater bed net use, various ways of increasing air flow in the net have been proposed. For instance, Briët et al. [38] studied the effect of making small solar-powered fans placed beside the net for cooling in the net, available to households in Accra, Ghana and found they were readily accepted and had a positive influence on net use. Taking a different approach, Knudsen et al. [39] proposed that even simple houses in tropical climates should be designed to include screened doors and windows on opposite walls to promote airflow. While these measures may have a cooling effect inside the bed net and thus modify human behaviour making bed net use more likely, this study suggests that the introduction of air movements will also modify mosquito behaviour around household nets, possibly to the detriment of the users. Re-focussing mosquito activity to the side of the net from the roof could, as discussed above, partially circumvent the benefits of certain ITN designs but becomes especially significant when the net is damaged. In a study of over 400 nets used over 3 years in Mozambique, Vanden Eng et al. [40] showed that, depending on net material (polyethylene or polyester) and hole size, 90–98% of holes were in the net sides. This is largely confirmed in another study of holes in ITNs from Malawi [41]. This would be problematic if cross-draughts introduced to enhance comfort in the net also re-oriented mosquitoes to the sides of the net where the greater abundance and size of holes will make net entry much more likely. Even for intact nets, cross-draughts might increase the chances for net users to be bitten if they are sleeping up against the net side. This could also make it more likely that the user will be bitten when they exit the net at night because they might be exiting into the swarming mosquitoes on the net side and mosquitoes concentrated on the net side might also be more likely to enter the net when the user exits, so that they are in the net when the unsuspecting user returns. In view of this, where there is a cross-draught present, it might be important to advise net users to exit and re-enter the net at night through the upwind side.

The wholesale diversion of household airflow could also affect vector control measures not directly related to bed nets. For instance, attraction of mosquitoes outside to insecticide-treated eave tubes relies on warm, odour-laden air from the house flowing upward and out through them [42]. This may be less likely if house modifications re-direct the host plume and its odours out through windows. In such cases, window traps [43] or sturdy window screens may be more effective.

Several initiatives have been undertaken to limit malaria and other tropical health risks through improved housing [44–46]. Evaluation of these measures usually includes objective measurements of indoor air movements [47], pest and insect entry, night-time temperature and humidity, etc. [39] and subjective evaluations of human responses with regard to perceived comfort levels, likelihood of using a net, preferences for different house designs [48]. However, these studies rarely investigate how the measures might affect vector behaviour with the potential result that some measures may have unintended negative consequences. The present research illustrates the importance of also including this analysis in house re-design initiatives aimed at malaria reduction.

## Conclusion

Recent research on mosquito behaviour around bed nets has led to new predictive models of mosquito net entry risk [18] and near-net behaviour [49] that could be used as tools in these assessments. This study is the first to investigate the dynamics of mosquito behaviour around bed nets in other than still air conditions and to look at the effects of ambient temperature on the mosquito-bed net interaction. While these results are laboratory-based and in need of further replication and validation in more complex real-world conditions, they suggest new features that could be incorporated into these models to further improve their accuracy and generalizability.

## Supplementary Information

**Additional file 1.** Clip of source video (.MP4) of appearances on net roof in fan-off leg of an HSCD session.

**Additional file 2. **Clip of video capture (.MP4) of approximately 12.5 min of Ethovision analysis output of the transition between fan off and fan on legs of a HSCD comparison run at 16 times normal speed (roof—upper panel, side—lower panel laid horizontally with ‘up’ to the left). Each red square represents a true mosquito appearance except at 27 s in both panels where a brief flash of light from within the net signalling the fan off-to-fan on transition creates dense accumulations of “appearances”.

## Data Availability

The datasets used during the current study are available from the corresponding author on reasonable request.

## Abbreviations

app.: Appearance near net
CO_2_: Carbon dioxide
CDC: Centers for Disease Control and Prevention
CPU: Central processing unit
HEPA: High efficiency particulate absorbing
HSCD: High-speed cross-draught
ITN: Insecticide-treated net
LSCD: Low-speed cross-draught
PVC: Polyvinyl chloride
